# Efficient Knock-in of a Point Mutation in Porcine Fibroblasts Using the CRISPR/Cas9-*GMNN* Fusion Gene

**DOI:** 10.3390/genes9060296

**Published:** 2018-06-13

**Authors:** Max Gerlach, Theresia Kraft, Bernhard Brenner, Björn Petersen, Heiner Niemann, Judith Montag

**Affiliations:** 1 Institute for Molecular and Cell Physiology, Hannover Medical School, Carl-Neuberg-Str. 1, 30625 Hannover, Germany; max.gerlach@stud.mh-hannover.de (M.G.); kraft.theresia@mh-hannover.de (T.K.); Brenner.Bernhard@mh-hannover.de (B.B.); 2 Institute of Farm Animal Genetics, Friedrich-Loeffler-Institut, Hoeltystrasse 10, Mariensee, 31535 Neustadt, Germany; bjoern.petersen@fli.de (B.P.); Heinrich.Niemann@fli.de (H.N.)

**Keywords:** genome editing, pig, CRISPR/Cas9, geminin, myosin, porcine fetal fibroblasts, SCR7

## Abstract

During CRISPR/Cas9 mediated genome editing, site-specific double strand breaks are introduced and repaired either unspecific by non-homologous end joining (NHEJ) or sequence dependent by homology directed repair (HDR). Whereas NHEJ-based generation of gene knock-out is widely performed, the HDR-based knock-in of specific mutations remains a bottleneck. Especially in primary cell lines that are essential for the generation of cell culture and animal models of inherited human diseases, knock-in efficacy is insufficient and needs significant improvement. Here, we tested two different approaches to increase the knock-in frequency of a specific point mutation into the *MYH7*-gene in porcine fetal fibroblasts. We added a small molecule inhibitor of NHEJ, SCR7 (5,6-bis((E)-benzylideneamino)-2-mercaptopyrimidin-4-ol), during genome editing and screened cell cultures for the point mutation. However, this approach did not yield increased knock-in rates. In an alternative approach, we fused humanized Cas9 (hCas9) to the N-terminal peptide of the Geminin gene (*GMNN*). The fusion protein is degraded in NHEJ-dominated cell cycle phases, which should increase HDR-rates. Using hCas9-*GMNN* and point mutation-specific real time PCR screening, we found a two-fold increase in genome edited cell cultures. This increase of HDR by hCas9-*GMNN* provides a promising way to enrich specific knock-in in porcine fibroblast cultures for somatic cloning approaches.

## 1. Introduction

The knock-in of specific mutations is essential for the generation of in vitro and in vivo models of inherited human diseases. Designer nucleases that introduce a double strand break at a specific locus in the genome have greatly advanced the development of such models [1]. The most commonly used designer nuclease is the Clustered Regularly Interspaced Short Palindromic Repeats (CRISPR) in conjunction with the associated Cas9-nuclease system (CRISPR/Cas9). CRISPR/Cas was originally discovered as part of the bacterial adaptive immune system [2], and its great biotechnological potential has emerged only recently [3,4,5]. The CRISPR/Cas9-system consists of a trans-encoded crRNA (tracrRNA) and the protospacer (crRNA) that binds via complementary base pairing to its 20 nucleotide long target DNA and recruits the CRISPR associated nuclease 9 (Cas9). Cas9 binds to the protospacer adjacent motif (PAM) on the target DNA and introduces a double strand break (dsbreak) at nucleotide 17 of the protospacer [6]. For experimental approaches, a chimeric single guide RNA has been produced by fusion of the crRNA and the tracrRNA that efficiently guides the Cas9 to the desired target locus to introduce dsbreaks [7].

The cell uses two different mechanisms to repair these dsbreaks; either non-homologous end joining (NHEJ) or homology directed repair (HDR). NHEJ is the predominant pathway and leads to insertions or deletions by unspecific ligation of the genomic DNA at the double strand break locus. HDR uses a donor DNA as a repair template and can be used to introduce specific genetic alterations. Whereas NHEJ is active during the whole cell cycle, HDR occurs mainly in the late S and the G2 phase and is thereby rather ineffective [8]. Therefore, HDR remains the bottleneck for the knock-in of disease causing point mutations in model animals [1].

Pierce and colleagues have shown that both pathways act concurrently and that reduction of NHEJ-activity increases HDR-rates [9]. Different studies have addressed the concurrent nature of NHEJ and HDR in order to increase the knock-in rates. NHEJ pathway inhibitors like SCR7 (5,6-bis((E)-benzylideneamino)-2-mercaptopyrimidin-4-ol) [10,11] or RS-1 [12] have been reported to increase HDR ratios significantly. SCR7 reduces the affinity of DNA-Ligase IV, the key enzyme of NHEJ-pathway, for dsbreaks [13]. It has been shown to increase the frequency of knock-in of point mutations and large DNA-fragments in cultured human cell lines [10,11]. For porcine fibroblasts the successful introduction of large fragments using SCR7 has been reported [14]. However, whether point mutations can also be introduced efficiently remains unknown.

Recently, two groups have reported a different approach. They generated fusion proteins of the humanized streptococcus pyogenes Cas9 (hCas9) and the N-terminal 110 amino acids of the human geminin gene (*GMNN*) [15,16]. Geminin is a regulator of the cell cycle by inhibiting DNA replication of associated genes [17]. Its concentration is increased in the S and G2-phase and decreases at the end of the M-phase, in the G1 phase it is not detected [17,18]. Fusion proteins with the N-terminal 110 amino acids are degraded in the late M and the G1 phase, respectively [19]. A fusion protein of Cas9 and Geminin would therefore only be active in the S and G2-phase, when HDR is dominant over NHEJ. With this approach, the knock-in rates could be increased in HEK293T (human embryonic kidney cells) [15] and induced pluripotent stem cells [16] in a gene locus dependent manner [16].

Here, we tested the effects of SCR7 and hCas9-*GMNN* fusion protein on the knock-in efficacy of a point mutation at the *MYH7*-locus in porcine fetal fibroblasts. Based on the CRISPR/Cas9 expression plasmid pX330 [3], we generated vectors that target the *MYH7*-gene in the porcine genome and express either the humanized Cas9 (hCas9) or the hCas9-Geminin fusion gene (hCas9-*GMNN*). We examined the knock-in efficacy comparing hCas9 with and without SCR7 and comparing hCas9 and hCas9-*GMNN*. Whereas SCR7 did not substantially increase genome editing efficacy, our results indicate that the use of Cas9-*GMNN* doubles the specific knock-in rate at the *MYH7*-locus in primary porcine fibroblasts.

## 2. Methods

### 2.1. Targeting Plasmids and Donor DNA

The plasmid pX330 for the single guide RNA (sgRNA) and the hCas9 protein were obtained from Addgene, Cambridge, USA (plasmid #42230). The design of sgRNAs was based on results from the Zhang laboratory website (http://crispr.mit.edu/). Sense and antisense oligonucleotides for three different sgRNAs specific for the *MYH7*-gene (723_3, 723_5, 723_9, Table 1) were synthesized by Biomers (Ulm, Germany) and hybridized by incubating 1 mM of each oligonucleotide in 8 mM Tris pH 7.5, 0.8 mM EDTA, and 40 mM NaCl for 30 min at 37 °C. The nucleotides were then denatured at 95 °C for 5 min and hybridized by subsequent cooling of 5 °C/min to 25 °C. Then, 50 pmol of the hybridized oligonucleotides were ligated to 5 µg of BbsI-restricted pX330 vector using T4-ligase (NEB, Frankfurt a.M. Germany) and amplified in *Escherichia coli.* DNA was isolated from single colonies and checked for correct introduction of the sgRNA via Sanger sequencing. The DNA was either used for transfection of porcine fibroblasts or used for further cloning. To fuse the *GMNN* peptide, a DNA fragment was synthesized (IDT, Leuven, Belgium) and ligated to pX330_*MYH7* vector via BsmI and EcoRI (Supplemental Material 2). The vector was amplified in *E. coli*, and DNA was extracted from single colonies, checked for correct introduction of the *GMNN* via Sanger sequencing, and used for transfection experiments.

### 2.2. Porcine Fetal Fibroblasts and Genome Editing

Primary porcine fetal fibroblasts used for genome editing of the *MYH7* gene were established from a male fetus at day 25 post conception and cultured for six passages as described previously [20]. For genome editing, 1 × 10^6^ cells were resuspended in 800 µL electroporation buffer (Bio-Rad, Munich, Germany), and supplemented with 65 pmol of ssODN (TGTCAATGAACTGGCCCTCGGGGATGGCCGCAGGGTTCAGGATGCCATACCTGGGGAGGAAGGTGCCTGGATTCACCCAAGTTCTGCATTG) encoding for the point mutation (underlined nucleotide) and 5 µg of the targeting plasmids pX330_723_Cas9-*GMNN* or pX330_723 respectively. Fibroblasts were transfected using the Gene Pulser XcelTM (Bio-Rad) at 250 V/400 µF and cultivated for 24 h at 37 °C. Subsequently, cells were either transferred to 6 well plates and cultivated for 6 days for surveyor nuclease assay or a total of 5 cells per well were cultured in 96 well plates for two weeks until confluency for screening assays. For SCR7-treatment, 1 µM SCR7 (Xcess Bioscience, San Diego, USA) was added to the media on day one and removed after 24 h, according to published protocols [10,11]. For lysis, 800 µL lysis buffer (0.02% sodium dodecyl sulfate (SDS), 0.05 mg/mL proteinase K, 1× PCR buffer S (Peqlab, Erlangen, Germany)) were added per six well or 50 µL per 96 well, respectively, incubated at 37 °C overnight and lysis was stopped by incubation at 95 °C for 12 min.

### 2.3. Surveyor Nuclease Assay

A *MYH7*-specific amplicon of 604 bp was amplified from 5 µL cell lysate of the 6 well plates in a PCR reaction mix (1× reaction buffer S, 12.5 mmol MgCl2, 5 pmol forward primer (TGGCAGGGCTAGGATCAGAAC), 5 pmol reverse primer (GATGGGAAGCAGTGCCATGT), 5 nmol dNTPs each, and 1 U Taq (PeqLab)) in a final volume of 50 µL. DNA was amplified by denaturation at 95 °C for 30 s, annealing at 68 °C for 45 s, and elongation at 72 °C for 30 s for 40 cycles. Subsequently, the target DNA was elongated at 72 °C for 2 min. For hybridization of mismatched strands, 12 µL PCR product were denatured at 95 °C for 10 min and the sample was cooled down to 85 °C by 2 °C per second and then to 25 °C by 0.1 °C per second. For surveyor nuclease assay (Transgenomic, Omaha, NE, USA), 1 µL each Surveyor Enhancer S and Surveyor nuclease S were added and incubated at 42 °C for 1 h and stopped by addition of 1.5 µL stop solution. Samples were analyzed on a 1.5% agarose gel. The expected fragments for genome editing induced mismatches were 328 bp and 276 bp for sgRNA CRISPR_3, 335 bp and 269 bp for sgRNA CRISPR_5 and two fragments of 302 bp for sgRNA CRISPR_9. Total editing was determined from the integrated optical densities (IODs) of the mismatch restriction bands and from the PCR products of 604 bp. IODs of each band were normalized to the respective size to correct for the intercalated ethidium bromide. These normalized IODs were used to calculate the total editing rate as the percentage of the restriction bands from the PCR product. Results were averaged from three independent transfections per sgRNA.

### 2.4. Screening for R723G-Genome Edited DNA

Mutation specific restriction analysis was performed by PCR-amplification of the genome edited locus using 1× reaction buffer S, 6.25 mmol MgCl2, 2.5 pmol forward primer (GTGCTGGAGGGCATCCGCATCT), 2.5 pmol reverse primer (CTTCTCTGCTCCTTTCCTGCTGTCAA), 10 nmol of each dNTP, and 1 U Taq (PeqLab) in a final volume of 12.5 µL. DNA was amplified by denaturation at 95 °C for 30 s, annealing at 62 °C for 45 s and elongation at 72 °C for 30 s for 40 cycles. Subsequently, the target DNA was elongated at 72 °C for 2 min. The PCR product was analyzed for introduction of the point mutation by BslI-restriction analysis as described previously [21]. For real time PCR screening we adapted a custom-SNP (single nucleotide polymorphism) assay (Thermo Fisher Scientific, Waltham, MA, USA). Mutant specific real time PCR was established using a plasmid encoding for the converter region of the *MYH7*-gene, either for the wildtype sequence or for the R723G mutation. The plasmids were mixed in defined ratios of R723G-/wildtype-DNA of 0%/100%, 10%/90%, 20%/80%, and 50%/50% (Figure S1A). For real time PCR, 1× TaqMan Gene Expression Master Mix (Thermo Fisher Scientific), 36 µM forward primer (GTGAATCCAGGCACCTTCCT, Biomers), 36 µM reverse primer (CCTTTCCTGCTGTCAATGAACTG, Biomers), 16 µM R723G-probe (6-FAM-TCAGGATGCCATACCT-BMN-535, Biomers), and 8 µM WT-probe (HEX-TCAGGATGCCATACCT-BMN-535, Biomers) and 2 µL of the plasmid or lysate in a final volume of 20 µL were subjected to 10 min activation at 95 °C and 40 successive cycles of 95 °C for 15 s and 56 °C for 45 s in a StepOne sytem (Thermo Fisher Scientific).

### 2.5. Sequence Analysis of R723G-Positive Cultures

Cell lysate (1 µL) was subjected to PCR reaction mix (1× reaction buffer S, 12.5 mM MgCl_2_, 5 pmol forward primer (GTGCTGGAGGGCATCCGCATCT), 5 pmol reverse primer (CTTCTCTGCTCCTTTCCTGCTGTCAA), 5 nmol each dNTP and 1 U Taq (PeqLab)) in a final volume of 25 µL. The solution was amplified by denaturation at 95 °C for 30 s, annealing at 62 °C for 30 s and elongation at 72 °C for 30 s for 40 cycles. Subsequently, the target DNA was elongated at 72 °C for 2 min. PCR amplicons for RNA quantification spanned 3 exons to discriminate between mRNA and potential genomic DNA contamination. The PCR product was purified using the PCR-clean-up kit (Macherey-Nagel, Germany) according to the suppliers’ instructions. Sanger sequencing was performed by GATC (Konstanz, Germany).

### 2.6. Data Analysis

Fluorescence signals of the real time PCR were analyzed using the StepOne Software v2.3 and Microsoft Excel according to the C_T_ method. To determine the threshold, mixtures of 10% R723G/90% WT and 0% R723G/100% WT plasmids were used as internal controls on every plate (Figure S1B). Successful knock-in was determined as amplification signal above the threshold. Graphical analysis was performed using Graph Pad Prism. Data are shown as mean ± standard deviation (SD). For statistical analysis, students *t*-test was applied. Significance was defined as a *p*-value < 0.05.

## 3. Results

**Figure 1:**
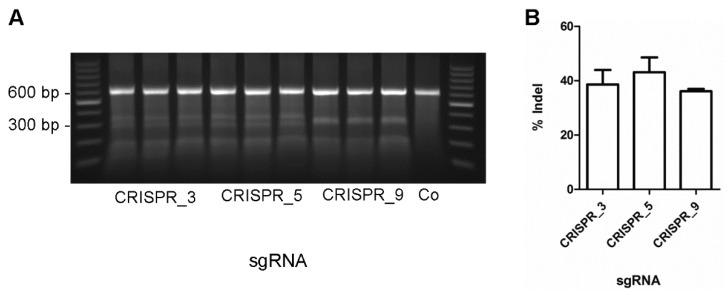
Total genome editing of *MYH7* gene. Three single guide RNAs (Clustered Regularly Interspaced Short Palindromic Repeats, CRISPR_3, CRISPR_5, CRISPR_9) were cloned into the pX330-vector. Vectors were transfected into porcine fetal fibroblasts in parallel experiments in triplicates. As a control, cells were subjected to the transfection protocol without CRISPR/Cas9 vector (Co). (**A**) A PCR amplicon of 604 bp of the *MYH7*-gene flanking the crRNA (CRISPR RNA, protospacer) binding site PCR product was generated. The strands of the PCR products were denatured, hybridized, and subjected to mismatch-dependent restriction analysis by surveyor nuclease. CRISPR/Cas9 induced mismatches would generate 328 bp and 267 bp fragments for CRISPR_3, 335 bp and 269 bp for CRISPR_5 and two fragments of 302 bp for CRISPR_9; (**B**) percentages of indels were calculated as the fraction of the mismatch-dependent fragments from the PCR product, each normalized to its fragment size to correct for intercalated ethidium bromide. The fraction of indels are depicted for each CRISPR-vector as mean ± standard deviation (SD).

The aim of this study was to develop a protocol to significantly increase the efficacy for knock-ins of the point mutation c.2167C > G in the *MYH7*-gene using porcine primary fibroblasts as a model system. Genome editing was performed by using the CRISPR/Cas9 expression vector pX330 [3] and introducing three different sgRNAs (CRISPR 723_3, CRISPR 723_5, CRISPR 723_9) that target the *MYH7*-gene at exon 23. The sgRNAs were introduced via BbsI-restriction sites into the chimeric guide scaffold to gain the pX330_723_3, pX330_723_5, and pX330_723_9 vectors and the sequence was verified. To test for the efficacy of our sgRNA, we analyzed the total editing rate using the surveyor nuclease assay. Transfected cells were harvested six days post transfection, the mutated locus was PCR-amplified and subjected to hybridization. Surveyor nuclease specifically restricts mismatched DNA, thereby detecting all mutations at the *MYH7* locus, including insertions, deletions, and point mutations. Since SNPs at this locus would also induce mismatches, we performed a control experiment using cells transfected without CRISPR/Cas9-DNA (Figure 1A, Co). These cells show no mismatch-derived restriction fragments, indicating that the fragments in the parallel experiments are due to CRISPR/Cas9 induced genome editing. We determined similar total editing rates of about 40% for each vector (Figure 1B). Note that the transfection efficiency was not taken into account. Even though we cannot exclude a bias from varying transfection efficacies, the highly comparable total editing rates from the three independent transfections per sgRNA indicate a minor influence of the transfection efficacy in these experiments. In summary, the results suggest highly comparable editing efficacies for the three different sgRNAs. CRISPR_723_3 introduces a double strand break two nucleotides upstream of the point mutation. Since double strand breaks close to the knock-in locus are supposed to provide the highest editing-efficacies, we chose this vector for specific genome editing experiments.

### 3.1. Effects of SCR7

**Figure 2:**
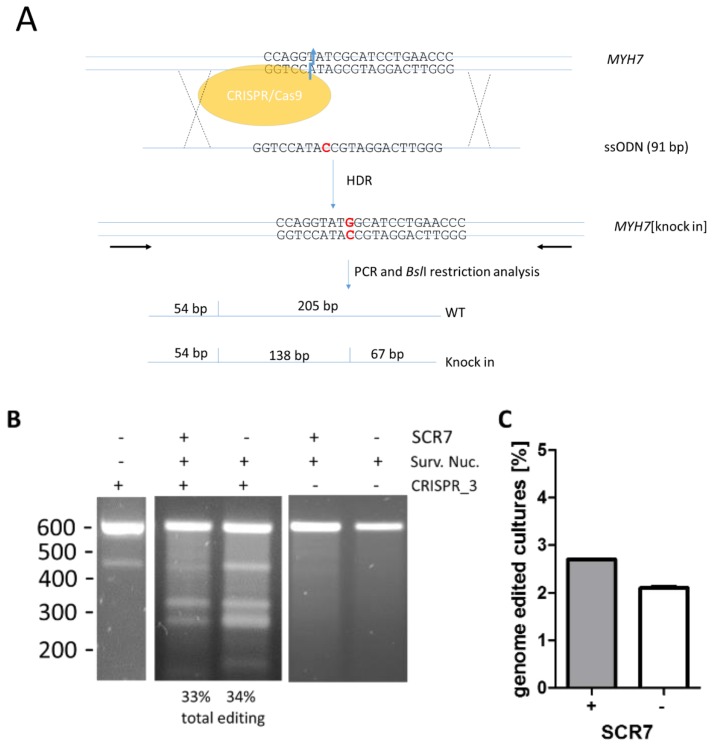
Effect of SCR7 on genome editing of the *MYH7* gene. (**A**) Schematic of the experimental setup. Porcine fetal fibroblasts were transfected with vector pX330 that encodes for hCas9 and the sgRNA CRISPR_3 (target site is indicated on the *MYH7*-gene) and a 91 bp single-stranded oligodeoxynucleotide (ssODN) representing the non-coding strand and the point mutation (indicated in red) at position 46. This point mutation generates an additional restriction site for BslI. In a PCR-based screening assay for successful genome editing, this restriction site generates a mutation specific 138 bp fragment which can be separated from the wildtype specific 205 bp fragment; (**B**) fibroblasts were transfected with the CRISPR/Cas9 vector pX330_723_3 (CRISPR_3 +) or without vector DNA (CRISPR_3 −) and were either treated with 1 µM SCR7 for 24 h post transfection (+ SCR7) or left untreated (− SCR7). To determine the total editing rate, a surveyor nuclease assay was performed. Fibroblasts without CRISPR/Cas9 vectors do not show mismatch-derived restriction bands of the expected size of 328 bp and 270 bp that are detected in CRISPR/Cas9-transfected cells. The total editing rates as the percentage of the normalized IOD of the 328 bp from the 604 bp bands for this specific experiment are given below the gel. The band at appr. 450 bp originates from the PCR (lane 1); (**C**) SCR7-treated and untreated cultures starting from 5 fibroblasts per well of 96-well plates were screened for knock-in of the point mutation c. 2167C > G by PCR and mutation specific restriction analysis. Percentages of cultures with at least one allele containing knock-in are depicted (*n* = 2 independent experiments).

To examine the effects of SCR7 on the knock-in efficacy in porcine fetal fibroblasts, in three independent experiments we co-transfected porcine fetal fibroblasts with the pX330_723_3 vector and a single-stranded oligodeoxynucleotide (ssODN) that encoded for the point mutation. The ssODN encoded for 91 bp of the non-coding strand of the *MYH7*-gene with the point mutation at position 46. No additional silent mutation or selection marker was introduced, since our study aimed at the knock-in of the single point mutation without further alterations in the gene. The point mutation generated a BslI-restriction site in the *MYH7*-gene that was used for screening of successful knock-in. A 259 bp amplicon was PCR-amplified and subjected to BslI-treatment. The wildtype allele generated two fragments of 205 bp and 54 bp. The knock-in allele generated three fragments of 138 bp, 67 bp, and 54 bp (Figure 2A).

Cells were incubated for 24 h in medium with or without SCR7 and total editing rates were confirmed as described above. No restriction fragments were generated if the cells had been transfected without CRISPR/Cas9-DNA in the presence or absence of SCR7. Therefore, we can rule out that the results are biased by SNPs at this locus (Figure 2B, lanes 2 and 3). Without SCR7, the average ratio of total genome editing was 30.7% ± 4.9% (*n* = 3 independent experiments). When cells were incubated with SCR7, a comparable fraction of 32.0% ± 1.7% was detected (*n* = 3; Figure 2B).

To examine the effect of SCR7 in cultures generated from the lowest cell count that allows proliferation, we seeded a minimum number of 5 cells per well in a 96 well plate. Again, 1 µM SCR7 was either added for 24 h or omitted. When cells reached confluency they were tested for the knock-in of the point mutation by BslI-restriction analysis and Sanger sequencing. We determined 2.7% positive cultures using SCR7 and 2.1% positive cultures without SCR7 (Figure 2C). Therefore, we could not detect a substantial increase in HDR using SCR7 in porcine fetal fibroblasts.

### 3.2. Effects of the Cas9-GMNN Fusion Protein

Recently, it was shown that fusion of Cas9 to the N-terminal amino acids of the *GMNN* protein can increase the rate of homology directed repair in immortalized cell cultures [15] and in human induced pluripotent stem cells [16]. Here, we directly compared the efficacy of the introduction of a point mutation into the porcine genome by Cas9 and a Cas9-*GMNN* fusion protein in fetal porcine fibroblasts. To facilitate screening, we used a real time PCR approach to identify cultures that contained genome-edited cells. The specificity of the assay was tested using plasmids encoding either the wildtype sequence or the mutated sequence in defined mixtures (Figure S1).

We generated an expression vector that encodes hCas9-*GMNN* based on the pX330_723_3 vector. A DNA-sequence encoding the first 330 bp of the *GMNN* gene was introduced (Supplemental Material 2) to generate the pX330_723_*GMNN* vector. The correct introduction of the *GMNN* was verified by sequencing. To compare the efficacy of genome editing in porcine fibroblasts, we co-transfected an ssODN donor encoding for the point mutation either with the Cas9-encoding pX330-vector or with the Cas9-*GMNN* pX330-vector. The transfected cells were cultivated and processed in parallel and under identical conditions.

**Figure 3:**
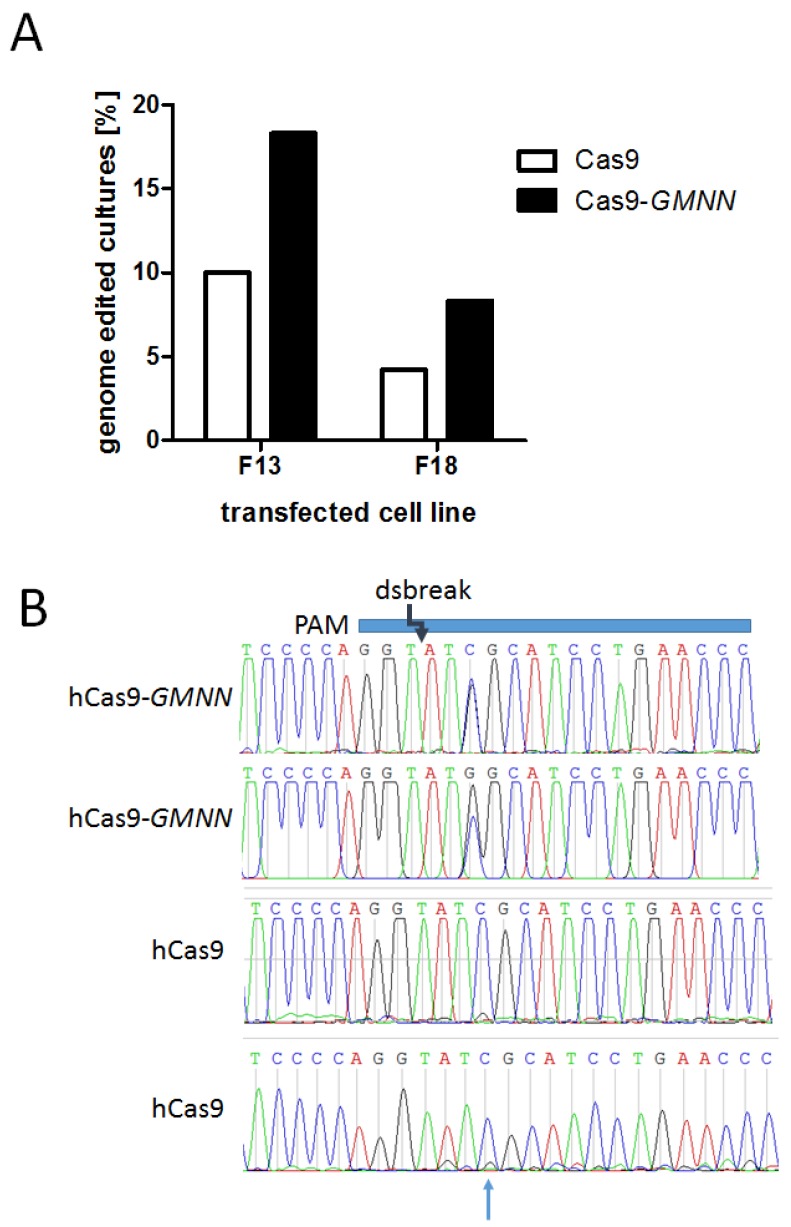
Efficacy of Cas9 and Cas9-*GMNN*-based knock-in in porcine fibroblasts. Porcine fetal fibroblasts were transfected in parallel experiments with ssODN encoding the point mutation R723G and CRISPR/Cas9 expression vectors either encoding for the Cas9 or the Cas9-*GMNN* fusion protein. The cells were plated at a density of 5 cells per well and cultivated until confluency in 96 well plates. (**A**) Cells were lysed and subjected to real time PCR screening for cultures with R723G-DNA. Two different fibroblast cell lines, F13 and F18, were transfected and screened for R723G-positive cultures. The percentage of R723G-positve cultures for the Cas9 and the Cas9-*GMNN* is plotted; (**B**) the genome edited locus of R723G-positive cultures was PCR-amplified and subjected to Sanger sequencing. Exemplary sequence chromatograms of two cultures each edited by hCas9-*GMNN* (upper panels) or hCas9 (lower panels), respectively, are shown. The protospacer adjacent motif (PAM) sequence, the protospacer (blue horizontal bar), and the position of the Cas9-induced double strand break are indicated schematically, the position of the point mutation is indicated by an arrow. Only hCas9-*GMNN* edited cultures showed high knock-in ratios of the point mutation C > G. Importantly, no additional alteration was detected at the double strand break locus. In contrast, hCas9 cultures show a substantially lower peak for the substituted G and additional modifications were detected.

The experiments were carried out in two different porcine primary fetal fibroblast cell lines. For cell line F13, 18 out of 180 cultures were positive for the point mutation using hCas9 and 33 out of 180 cultures were positive using hCas9-*GMNN*. For cell line F18, 4 out of 95 cultures were positive for the point mutation using hCas9 and 7 out of 84 using hCas9-*GMNN* (Table 2). Even though the total genome editing efficacy varied between the two fibroblast cell lines, both showed substantially increased ratios of genome editing using the Cas9-*GMNN* fusion gene (Figure 3A). On average, the ratio of mutation positive cultures was increased by 1.9 ± 0.1-fold (student *t*-test, *p* < 0.05) using the Cas9-*GMNN* fusion protein (Table 2).

We chose 10 R723G-positve cultures and checked for correct knock-in by Sanger sequencing. Even though sequence analysis showed that most clones only contained low levels of R723G-DNA, two clones had almost equal levels of R723G and WT peak intensities and no indels could be detected at the knock-in locus. In contrast, cultures genome edited by Cas9 alone showed only low ratios of mutant mRNA and in some cases also additional modifications (Figure 3B).

## 4. Discussion

In the past years, tailored endonucleases such as zinc finger nucleases, Transcription Activator-Like Effector Nuclease (TALENs), and the CRISPR/Cas9 system have emerged as important tools for genome editing of large animals [1]. This raises hopes for the efficient generation of large animal models carrying mutations causative for human diseases for investigating etiology and new therapies.

The goal of this study was to increase the frequency of specific point mutations in porcine fibroblasts for use in somatic cell nuclear transfer (SCNT) based cloning. The NHEJ-based knock-out generation and selection-marker supported HDR-based introduction of mutations in the porcine genome is quite efficient [1]. In contrast, the introduction of a single point mutation using designer nuclease-based genome editing is very inefficient, and the efficacy depends on the respective cell type [22]. Porcine fetal fibroblasts have been used successfully for genome editing followed by SCNT-based cloning, both for knock-outs [23,24,25] and also for knock-ins [21]. Nevertheless, total knock-in efficacy remains low in these cells, for an efficient production of genome edited pigs via SCNT, this ratio needs to be improved.

Several different studies report an increase of the HDR ratio using the NHEJ pathway inhibitor SCR7. SCR7 inhibits DNA ligase IV, the central ligase of NHEJ-driven DNA repair. It was therefore supposed to be a potent inhibitor of NHEJ [13]. Due to the concurrent action of NHEJ and HDR, the inhibition of NHEJ was assumed to promote HDR [11]. Indeed, initial reports claim an increase in HDR-rates of 4–5% up to 19% in cultured human cell lines [10,11]. A study on the insertion of GFP into herpes simplex virus-1 DNA in HEK293T cells confirms these findings showing a more than 10-fold increase of HDR and a decrease of NHEJ [26]. However, the majority of studies using SCR7 determined substantially lower HDR rates. In HEK293T-cells [15], HEK293A cells [27], rat zygotes [28], MCF-7 and HCT-116 cells [29], and in porcine fetal fibroblasts [14], the increase was no more than 2-fold. In addition, another study even showed that the effect of SCR7 on HDR was negligible in human pluripotent stem cells. The authors assume that in pluripotent cells, the inhibition of the canonical DNA ligase IV-dependent NHEJ pathway leads to the activation of non-canonical NHEJ pathways, finally diminishing the SCR7 effect [30]. Also in our study, we show no major effect of SCR7 on knock-in efficacy in porcine fetal fibroblasts. Therefore, non-canonical NHEJ pathways may also act in the fetal fibroblasts. In line with this assumption, the study from Li and colleagues using SCR7 in porcine fetal fibroblasts showed a substantially lower increase of HDR [14] as compared to cultured cell lines [10,11,29]. Nevertheless, in contrast to our study, Li et al. described a two-fold increase in HDR by SCR7 in porcine fetal fibroblasts [14]. They used ten to 200-fold higher concentrations of SCR7, which might have also inhibited non-canonial NHEJ pathways in the cells. However, concentrations of SCR7 higher than 1 µM reduce cell growth by inhibiting entrance into S/G2 phase [11]. Since our cell line shows low proliferation capacities, we chose to keep the SCR7 concentration at 1 µM. This concentration successfully increased HDR in other studies [26,27,28,29]. A second reason for low effect of SCR7 in our study might be that our experimental setup aimed at the introduction of a single point mutation without further alterations of the genomic locus. Therefore, we could not generate an additional silent mutation in the PAM. Our ssODN introduces an alteration in the crRNA binding site but none in the PAM. Therefore, successfully altered alleles may be re-cut in subsequent cell cycles as long as Cas9-protein is produced, although with decreased efficacy due to the mutation in the crRNA binding site. Indels in NHEJ-edited alleles will generate more pronounced alterations of the crRNA binding site than HDR-introduction of a point mutation and thereby prevent CRISPR/Cas9 binding. Thus, re-cutting will occur more often in HDR-corrected alleles. The other studies using SCR7 disrupt the PAM-sequence [14,15,27,28,29] and thereby prevent re-cutting. Therefore, re-cutting events might have diminished the potential increase in HDR by SCR7 on porcine fibroblasts [14] in our experimental setup. In summary, we assume that the advantageous effect of SCR7 on HDR cannot be generalized for all knock-in strategies but needs to be determined for each individual setup. The results from our and other studies suggest that SCR7 is most likely not effective if the editing design does not prevent re-cutting [14] and in cell types which activate non-canonical NHEJ pathways [30].

Since the inhibition of NHEJ using the small molecule inhibitor SCR7 was not successful in our setup we aimed at a different approach. Geminin, a regulator of gene expression, is degraded in the G1 phase of each cell cycle [17]. Sakaue-Sawano and colleagues first detected its biotechnological value for visualization of the cell cycle by fusion to fluorescent proteins. They also found that the N-terminal 110 amino acids contain the cell cycle dependent degradation signal [19]. In 2016, two groups independently applied the *GMNN* fusion for direct cell cycle dependent control of protein expression, namely of Cas9 [15,16]. Previous studies had shown that the arrest of cells in the M- or S-phase during Cas9-delivery increased the HDR ratio, whereas arrest in G1 increased NHEJ [26]. Therefore, it was postulated and proven that the rapid and effective degradation of Cas9 in the G1-phase by fusion to the degradation peptide of *GMNN* also increases HDR rates since the Cas9-*GMNN* protein has the highest concentration in the S and G2 phase, when the HDR pathway is more active than NHEJ [15,16]. Gutschner and colleagues transfected the pX330 vector that encodes the Cas9-*GMNN* fusion gene to insert 20 bp at an endogenous locus in HEK293T cells. Their approach increased the knock-in efficacy about 1.5-fold (from 9.7% to 13.8%). An additional cell cycle arrest further increased this rate to two-fold [15]. Howden and colleagues used a slightly different approach and transfected Cas9-*GMNN* mRNA either to introduce GFP in human fibroblasts or to substitute four nucleotides—three in the crRNA binding site and one in the PAM—in induced pluripotent stem cells. In this study, the total number of knock-ins was increased approximately 1.75-fold (from 9.7% to 16.6%) [16]. Also in our setup, using porcine fetal fibroblasts we showed that the HDR-mediated introduction of a point mutation was increased from on average 8% to 15.2% of genome edited cultures at the *MYH7* gene locus. This represents a 1.9-fold increase. These fibroblast cultures had been generated from five fibroblasts plated per well after transfection and cultivated until confluency. Presumably, in most positive cultures, only one out of the five cells had been successfully genome edited and most likely on one allele only. Thus, the total knock-in efficacy per cell number is likely substantially lower. Nevertheless, in direct comparison with hCas9, the hCas9-*GMNN* fusion protein leads to an almost 2-fold increase in successfully genome edited cultures. In addition, as Sanger sequencing results suggest the number of genome-edited cells per culture was increased by hCas9-*GMNN*, which will be essential for SCNT-based cloning of pigs with a specific knock-in.

Whereas Gutschner and colleagues described increased HDR rates and unchanged NHEJ [15], Howden and colleagues also show that Cas9-*GMNN* substantially reduces the rate of additional NHEJ-based mutations at the knock-in locus, especially in human fibroblasts [16]. They assume that this is due to the administration of Cas9-*GMNN* mRNA instead of plasmid, which would reduce the amount of expressed Cas9 molecules in consecutive cell cycles [16]. Even though we did not perform extensive analysis on additional mutations, Sanger sequencing results show substantial levels of NHEJ-based additional modification in Cas9 but not in Cas9-*GMNN*-derived cultures. This also indicates that transfection of Cas9-*GMNN* as a plasmid can reduce NHEJ rates. We suppose that, in addition to the administration of Cas9-*GMNN* as mRNA or even as protein, an efficient NHEJ reduction will also depend on the transfected cell type. The relative time cells spend in the G1 phase differs from cell type to cell type. In HEK293T cells used by Gutschner and colleagues, the G1 phase is rather short, with only 1/3 of the cells being in G1 at any specific point in time [31]. Mammalian fibroblasts, in contrast, can adopt a quiescent state the G1 phase from which they switch to proliferation [32]. Here, 50–92% of the cells can be in G1 at once [33]. Therefore, the effect on NHEJ, which is predominant in G1, will be more pronounced in fibroblasts as used in Howdens and our study, than in HEK293T cells. In accordance with this, Howden and colleagues found that human induced pluripotent stem cells provide a lower NHEJ reduction than human fibroblasts. They also assume that this is due to the shortened G1/S phase of pluripotent stem cells [16].

It should be noted that the genome editing efficacy could have been further increased by the use of ssODNs with additional silent mutations in the PAM sequence as also discussed for SCR7. However, in our approach, this was not applicable. The general incidence of re-cutting is most likely higher in cells transfected with hCas9, which is active over the whole cell cycle as compared to hCas9-*GMNN*. Therefore, an ssODN which introduces mutations in the PAM would presumably increase the general knock-in efficacy more for hCas9 than for hCas9-*GMNN*. This may slightly reduce the positive effect of hCas9-*GMNN* in direct comparison to hCas9. In turn, the use of Cas9-*GMNN* protein for transfection might also reduce re-cutting events for our approach, since the whole Cas9 protein would be degraded after one cell cycle. Further studies using expressed Cas9-*GMNN* may therefore reveal even higher knock-in rates. A further increase may be achieved by arresting the cells in the S phase using nocodazole [15]. However, whereas such an arrest has been shown to substantially increase HDR in immortalized cells, primary cell cultures remained mainly unaffected by nocodazole [26,34]. Recent studies have also shown that the location of the point mutation within the ssODN can increase the HDR incidence [35]. A combination of Cas9-*GMNN* with specially designed ssODN and/or cell cycle arrest in the S-phase may further increase the knock-in efficacy in porcine fetal fibroblasts and thereby facilitate the production of genome-edited pigs.

## 5. Conclusions

In summary, we provide compelling evidence for an increase of homology directed repair by hCas9-*GMNN* directed genome editing in porcine fetal fibroblasts. The second approach tested to increase HDR with SCR7 was not successful. Our results show that use of hCas9-*GMNN* increases the knock-in of a single point mutation in the crRNA-binding site more effectively than the addition of SCR7. This may indicate that the coordinated expression of Cas9 in HDR-dominated cell cycles is more effective than the inhibition of NHEJ-associated DNA ligase IV, at least in mammalian fibroblasts. This assumption is supported by a study from Yang and colleagues showing that cell cycle arrest using Nocadozole or ABT-751 more effectively increases HDR than the use of SCR7 [30].

The generation of genome-edited large animal models, e.g., for inducing human mutations to create new models of human diseases, critically depends on a high efficacy of HDR. The results presented here demonstrate that hCas9-*GMNN* provides a highly promising, easily applicable method for knock-in generation in porcine fibroblasts.

## Figures and Tables

**Table 1:** *MYH7*-specific single guide RNAs.

sgRNA	Sequence
723_3	AGGGTTCAGGATGCGATACCTGG
723_5	TGCGTGGCCTTAGATTCTGTGGG
723_9	GATTCACCCAAGTTCTGCATTGG

**Table 2:** Screening of cell cultures for successful genome editing.

Fibroblasts	Cas9			Cas9-*GMNN*		
	Analyzed Cultures	Genome Edited Cultures		Analyzed Cultures	Genome Edited Cultures	
		Absolute	Percent		Absolute	Percent
PFF F13	180	18	10.0%	180	33	18.3%
PFF F18	95	4	4.2%	84	7	8.3%
Total	275	22	8.0%	264	40	15.2%

## Supplementary Materials

The following are available online at http://www.mdpi.com/2073-4425/9/6/296/s1. Figure S1: Realtime PCR based screening assay for the R723G knock-in and DNA-fragment used for cloning of GMNN.
