# Supplementary material for: Efficient Knock-in of a Point Mutation in Porcine Fibroblasts Using the CRISPR/Cas9-GMNN Fusion Gene
Source: Genes (Basel). 2018 Jun 13;9(6):296. doi: 10.3390/genes9060296 (PMC6027509; doi:10.3390/genes9060296)
Supplement: Supplementary file 1 [file genes-09-00296-s001.docx]

**Selection-independent *knock-in* of a point mutation in porcine fibroblasts using the CRISPR/Cas9-*GMNN* fusion gene**

Gerlach M, Kraft T, Brenner B, Petersen B, Niemann H, Montag J

**Electronic supplemental material**

**Realtime PCR Assay**

The specificity of the Realtime PCR assay was tested using plasmids encoding for either the wildtype sequence or the mutated sequence in defined mixtures of 0%/100%, 10%/90%, 20%/80% and 50%/50% of mutant/wildtype DNA. The ratios encompass the expected low rate of mutant mRNA in the cultures of porcine fetal fibroblasts. The fibroblasts were seeded at 5 cells per well in 96-well plates, the lowest cell count that allowed proliferation of the cells. If a mono-allelic genome editing in one out of five cells occurs, it would result in 10% of mutant alleles. Our assay specifically and reliably detects fractions of at least 10% mutant DNA, whereas no unspecific detection of wildtype DNA (0% R723G/100% WT) was found (Figure S1A). The standard plasmid mixtures for 10% R723G/90% WT and 20% R723G/80%WT were used as internal controls in each assay to determine R723G-positve cultures (Figure S1B). However, it should be noted that a quantification of the genome editing rate within one culture is not possible using this assay.

**Figure S1:**


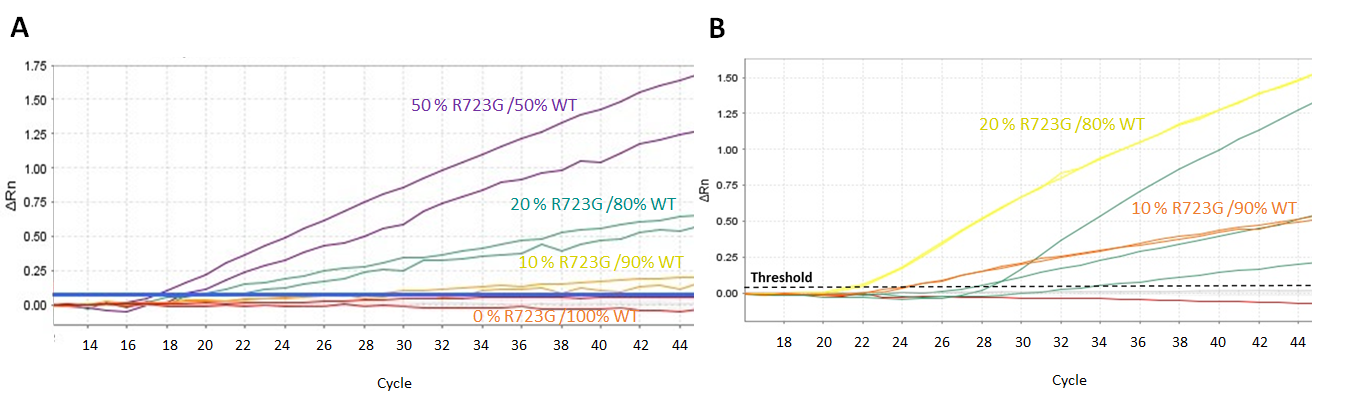


**Figure S1: Realtime PCR based screening assay for the R723G *knock-in***

A SNP-detection assay with mutation and wildtype specific probes was used to identify the R723G DNA in the samples. **(A)** As test for the detection limit of the mutant and the wildtype sequence, standard plasmids that encode for the wildtype and mutant R723G-locus of the *MYH7*-gene were mixed in defined ratios (0% R723G/100% WT, 10% R723G/90% WT; 20% R723G/80% WT, 50% R723G/50% WT) and 10^7^ copies were subjected to the Realtime PCR assay for 45 cycles. Signals for 10%, 20% and 50% R723G DNA were above the threshold whereas with 0% R723G DNA no signal above threshold was gained. **(B)** Exemplary analysis of the mutant R723G-locus in porcine fibroblast cultures. The cell cultures were lysed and 1 µl of the lysate was subjected to real-time PCR. A total copy number of 10^6^ standard plasmid mixtures (10% R723G/90% WT in orange and 20% R723G/80% WT in yellow) were used as internal controls. Cultures with signals above the threshold are indicated in green and were defined as positive for R723G-DNA. An exemplary negative culture with a signal below the threshold is depicted in red.

**DNA-fragment used for cloning of GMNN.**

A DNA fragment encoding for the 3’-end of the hCas9 in the pX330 vector backbone (black letters) and the nucleotides 1-330 of the GMNN gene (red letters) was synthesized (IDT, Leuven Belgium). The fragment was treated with restriction enzymes BsmI (restriction site is marked in yellow) and EcoRI (restriction site is marked in green) and cloned into the pX330-vector.

CGGCCGGAAGAGAATGCTGGCCTCTGCCGGCGAACTGCAGAAGGGAAACGAACTGGCCCTGCCCTCCAAATATGTGAACTTCCTGTACCTGGCCAGCCACTATGAGAAGCTGAAGGGCTCCCCCGAGGATAATGAGCAGAAACAGCTGTTTGTGGAACAGCACAAGCACTACCTGGACGAGATCATCGAGCAGATCAGCGAGTTCTCCAAGAGAGTGATCCTGGCCGACGCTAATCTGGACAAAGTGCTGTCCGCCTACAACAAGCACCGGGATAAGCCCATCAGAGAGCAGGCCGAGAATATCATCCACCTGTTTACCCTGACCAATCTGGGAGCCCCTGCCGCCTTCAAGTACTTTGACACCACCATCGACCGGAAGAGGTACACCAGCACCAAAGAGGTGCTGGACGCCACCCTGATCCACCAGAGCATCACCGGCCTGTACGAGACACGGATCGACCTGTCTCAGCTGGGAGGCGACATGAATCCCAGCATGAAGCAGAAACAGGAAGGAATCCAAGAGAATGTAAAGAGTAGTCCTGTTCCAAGAAGAACTCTGAAGATGATTCAGCCTTCTGTGGCTGGATCTCTTGTTGGAAGAGAAAATGAGTCGGTTAAAGGCTTGTCCAAAAGGAAACAATGGAGTGACCAGTCAATATCAAAGACTTCCGGCTCTGGAGTTCTTACTGTCCCAGAACATAGTGAAAATAAAAATGTTGGAGGAGTGACCCAAGAAGCATTTGATCTTATGATTACAGAAAATCCATCCTCTCAATATTGGAAAGAAGTGGCAGAAAAACGGAGGAAGGCTTAAGAATTCCTAGAGCTCGC
